# Iodine Rendered Soluble by Syrup of Orange-peel and Tannin

**Published:** 1852-02

**Authors:** 


*Iodine rendered soluble by Syrup of Orange-peel and Tannin.*—M. Debauque mentions, in the *Journal de Pharmacie* of Antwerp, that he has found means of keeping iodine in a state of solution, when added to mixtures in the form of tincture. The author uses for that purpose, syrup of orange-peel, which answers the purpose perfectly. It was suspected that *tannin* was mainly instrumental in this result; and this was rendered evident by putting a few grains of tannin into a quantity of water to which tincture of iodine has been added, and in which the iodine had of course been precipitated. The addition of the tannin caused the iodine to be immediately re-dissolved. Thus will the syrup of orange-peel be advantageously added to mixtures containing tincture of iodine, and tannin to injections composed of water and the same tincture.—*Lancet.*

